# *McWRI1*, a transcription factor of the AP2/SHEN family, regulates the biosynthesis of the cuticular waxes on the apple fruit surface under low temperature

**DOI:** 10.1371/journal.pone.0186996

**Published:** 2017-10-26

**Authors:** Suxiao Hao, Yiyi Ma, Shuang Zhao, Qianlong Ji, Kezhong Zhang, Mingfeng Yang, Yuncong Yao

**Affiliations:** 1 Beijing Key Laboratory of New Technology in Agriculture Application, Plant Science and Technology College, Beijing University of Agriculture, Beijing, China; 2 College of Biological Science and Engineering, Beijing University of Agriculture, Beijing, China; 3 College of Landscape Architecture, Beijing University of Agriculture, Beijing, China; 4 Beijing Collaborative Innovation Center for Eco-environmental Improvement with Forestry and Fruit Trees, Beijing, China; Key Laboratory of Horticultural Plant Biology (MOE), CHINA

## Abstract

Cuticular waxes of plant and organ surfaces play an important role in protecting plants from biotic and abiotic stress and extending the freshness, storage time and shelf life in the post-harvest agricultural products. WRI1, a transcription factor of AP2/SHEN families, had been found to trigger the related genes taking part in the biosynthesis of seed oil in many plants. But whether WRI1 is involved in the biosynthesis of the cuticular waxes on the *Malus* fruits surface has been unclear. We investigated the changes of wax composition and structure, the related genes and WRI1 expression on *Malus asiatica* Nakai and *sieversii* fruits with the low temperature treatments, found that low temperature induced the up-regulated expression of *McWRI1*, which promoted gene expression of *McKCS*, *McLACs* and *McWAX* in very-long-chain fatty acid biosynthesis pathway, resulting in the accumulation of alkanes component and alteration of wax structure on the fruit surface. Corresponding results were verified in *McWRI1* silenced by VIGS, and *WRI1* silenced down-regulated the related genes on two kinds of fruits, it caused the diversity alteration in content of some alkanes, fatty acid and ester component in two kinds of fruits. We further conducted Y1H assay to find that McWRI1 transcription factor activated the promoter of *McKCS*, *McLAC* and *McWAX* to regulate their expression. These results demonstrated that McWRI1 is involved in regulating the genes related synthesis of very long chain fatty acid on surface of apple fruits in storage process, providing a highlight for improvement of the modified atmosphere storage of apple fruits.

## Introduction

Cuticular waxes is a class of organic compounds on plant surfaces to isolate plant and its environment as a barrier, which are believed to have many functions, including limiting the transpiration water loss [1], reflecting UV-radiation [2], providing self-cleaning mechanism [3], preventing pathogens and insect infection [4–7]. It has increasingly been paid attention to play an important role in the fresh and storage of agricultural products and extending the shelf life.

Cuticular waxes are consist of very-long-chain fatty acid (C20 to C34) and their derivatives, which contain wax esters, alkanes, aldehydes, ketones, alcohols and secondary alcohols [8]. Belding and his colleges dissected fruit cuticular waxes of 18 classes Malus plants by GC-MS and found there are 40%-70% alkane in aliphatic compound, of which nonacosane is dominant [9]. Moreover, these chemical components are demonstrated, to some extent, to be associated with morphology of cuticular wax [10]. In most plants surface, three types morphology have been concluded with accordant chemical component, they are tubular structure which consist of nonacosane and its homologues [11], tubular structure consist of ketone [12, 13] and laminar structure which consist of multiple components [14]. Derased cuticular wax is composed of majority alkanes. However, the microscopic observation indicated the environment stress could decrease the cuticular wax content and composition, resulting in dictyoid crack in the fruit surface [15, 16]. These microscopic changes are not only affected by composition of lipids, which is regulated by the gene expression and transcript level in the cuticular wax biosynthesis signal pathway, but also are enslaved to the environmental condition, including light, temperature and humidity during fruit development and in postharvest process [17].

The cuticular wax biosynthesis on the surface of the most plant is considered by researchers includes four steps. The first step is C16 and C18 fatty acids synthesized in the plastids, the second step is the biosynthesis of very long chain fatty acid, the third step is producing wax component by utilizing the products of last step through acyl reduction pathway or removing carbonyl way, the fourth step is exporting the components to the cuticular wax layer of plant by the co-operation of ATP binding cassette transporter and lipid transfer protein [18]. These steps can be regulated partly by several key genes, such as *KCS*, *LACS*, *PKM2*, *KPHMT*, in their branches of biosynthesis. There, *KCS* gene encodes a 3-ketoacyl-CoA synthase, and act as the first component of the elongation complex, which involved in synthesis of very long chain fatty acid, influencing the chain length of substrates and products [19, 20]. Long-chain acyl-CoA synthetases (*LACSs*) catalyze the conversion of free fatty acids to fatty acyl-CoAs [21], A recently study indicate that an acyl-CoA synthetase encode gene, *LACS2*, take part in cuticular wax biosynthesis [22], and also a crucial regulatory enzyme in glycolytic, pyruvate kinase (*PK*), can catalyze the irreversible synthesis of pyruvate and ATP from phosphoenolpyrtvate and ADP, glycolysis transforms sugars to precursors for fatty acid synthesis. What's more, there is a certain relationship in the *wri1* mutant of Arabidopsis between glycolysis and seed metabolism [23]. *WAX* is associated with wax produced and cuticle membrane, some studies revealed that *W1*, *W2* and *W3*, several homologous genes to *WAX*, were involved in the wax production in wheat [24–26]. Ketopantoate hydroxymethyl transferase (*KPHMT*) catalyzes, the first step of the pantothenic acid biosynthetic pathway, may be the precursor of coenzyme and enzyme co-factors essential for crucial fatty acid metabolism [27]. At present, it is still rare molecule mechanism of joint action and transcript regulation these genes participate in the cuticular wax synthesis on the surface of plants and horticultural fruits.

*WRI1* (*WRINKLED1*) is a transcription factor of AP2/SHEN families that is response to ethylene, which may trigger the genes taking part in the biosynthesis of fatty acid. Studies have shown that in Nicotiana benthamiana, proteasomal degradation of WRI1 resulted in the accumulation of fatty acids decreased [28]. Similarly, expression of camelina WRINKLED1 isoforms rescued the seed phenotype of the Arabidopsis wri1 mutant and increased the triacylglycerol content in tobacco leaves, which further increased fatty acids in biomass [29]. WAX INDUCER1 (WIN1)/SHINE1 (SHN1) can regulate cuticle development in Arabidopsis and torenia fournieriand its phylogenetic neighbors in the AP2/ERF family [30]. In *Arabidopsis* thaliana plants of *AtWIN1*/*SHN1* over-expression, the wax content of stem and leaf epidermis is increased by 4.5 folds compared with wild type, with that, some wax synthesis related genes, *KCS1*, *LACs*, *CER1*, *CER2*, raised expression. These indicated that *WIN1*/*SHN1* can regulate expression of the wax synthesis related genes, and improve wax accumulation, resulting in enhance the drought resistance [31]. All of above studies indicate that *WIN1 / SHN1* plays an important role in *WAX* biosynthesis on surface of plants by regulating wax synthesis related genes, however, the regulation mechanisms of WRI1 in *WAX* biosynthesis and relative genes on the surface of horticultural fruits is still unclear.

Low temperature is recommended storage to protract fruit postharvest life [32]. In *Arabidopsis* under low temperature, the density, shape and size of wax crystalloids on stem of mutants and WT changed. Wax crystal structures on WT fused to big horizontal plates, greatly covering the surface of stems, which might help protect plant from cold hardness and decrease water loss [33]. In this study, *Malus* crabapples fruits were used as material as its valuable functions. We have compared different appearances and gene expression levels among low temperature stimulated, transient silencing lines and control group. Furthermore, HPLC analysis provided an insight into the accumulation of wax compounds in the different treated group. qRT-PCR analysis showed that the levels of expression of these genes were related to wax accumulation, especially the *WRI1* gene.

## Materials and methods

### Plant materials and growth conditions

Malus crabapples cultivar, *M*.*cv* ‘Royalty’, was used to gene isolation. And the four representative varieties, *Malus asiatica* Nakai ‘binzi’, *Malus prunifolia*, *Malus asiatica* Nakai and *Malus sieversii*, were used for further research. All of these six-year-old trees grafted on *Malus* sp. Hupehensis were grown in the Crabapple Germplasm Resources Nursery of Beijing University of Agriculture.

Fruit peels of *M*.*cv* ‘Royalty’ were collected at the fruit mature stage (on August 23) and thirty fruits of each of the four Malus cultivars were harvested in the beginning of September for the further assays.

Temperature treatments were conducted on the fruits at 4°C (low temperature) and 25°C (normal temperature as CK) for 15 days. All samples were frozen in liquid nitrogen and stored at -80°C to further tests and analyzed in triplicate.

### Isolation of full-length *McWRI1* cDNA, sequence comparison, and phylogenetic tree construct

Total RNA of fruit peel in *M*.*cv* ‘Royalty’ was extracted using a EASYspin Plus plant RNA kit and RNAprep Pure plant kit (Biomed, China), following the manufacturer's instructions, then treated with DNase I (TaKara, Japan) at 37°C for 30 min. The RNA samples (1μg) were reverse transcribed into complementary DNA (cDNA) using an oligo (dT) 18 primer and M-MLV reverse transcriptase (TaKaRa) following the manufacturer’s protocol. A 10 μL aliquot of cDNA was diluted to 100 μL with RNase-free water, and 2 μL (50 ng) of the diluted cDNA was used for PCR.

Full-length cDNA of *McWRI* was obtained by PCR using the primers of McWRI1-F/R (S1 Table). The full-length cDNA sequence was used to search homologous sequences via the National Center for Biotechnology Information BLASTX. Multiple alignments of amino acid sequences were performed using DNAMAN 7. A phylogenetic tree was then constructed by the neighbor-joining method with MEGA 7.

### Vector construction and agroinfiltration in fruits

The cDNA of *McWRI* (without TAG) was cloned into the expression vector pTRV using the primer pair McWRI2-F/R (S1 Table). The resultant vector was named pTRV-McWRI.

Thep TRV-McWRI was transformed into Agrobacterium tumefaciens GV3101. After Agrobacterium tumefaciens grew to saturation in Luria-Bertani medium, the culture was centrifuged. Then the pellet was resuspended in 10 mM MES, 10 mM MgCl2 and 200 μM acetosyringone solution to a final optical density of 1.0 at 600 nm and then incubated at room temperature for 3–4 h without shaking. Before infiltration, A. tumefaciens cultures containing pTRV1 and pTRV-McWRI were mixed in a 1:1 ratio. And pTRV1 and pTRV empty vector were mixed as control. The whole fruits were infiltrated and kept in the dark at 4°C for 7 days. Subsequently, these fruits were maintained for an additional 3–5 days under 24 h of continuous white light (200 μmol/m2/s) at 17°C in a growth chamber. The transient expression assays were repeated at least three times, and at least thirty fruits were used in each experiment [34].

### GC-MS analysis

Putting fruit peel samples with same treatment in 3 different 1000ml beakers for 30s to dewaxing, each beaker contain 300ml chloroform and 10 same treated samples, then take all the chloroform together, repeat 3 times. In order to qualitative analysis the component of the different treat in variety varieties, use TurboMassVer 5.4.2 software to deal with the chemistry information. The above extractives were analyzed by online linked gas chromatograph/mass spectrometer (GC-MS), respectively. The GC/MS analysis was carried out on an Aglient 6890 N + 5975C GC-MSTM (Aglient Co., Ltd, USA), which was linked to a mass selective detector. An elastic quartz capillary column DB-5MS (30 m × 250 μm × 0.25 μm) coated with a neutral phase (hewlett–packard-5 cross-linked 5% phenyl methyl silicone) was used. The carrier gas was helium and the injection port temperature was 250°C. The temperature program of GC began at 50°C and increased at the rate of 8°C/min until 250°C, 5°C/min until 300°C was reached, followed by a split injection at a ratio of 15:1. The program of MS was scanned over the 35–335AMU (m/z) respectively, with an ionizing voltage of 70 eV and an ionization current of 150 μA of electron ionization (EI). The flow velocity of helium was 1.2 ml/min. Ion source temperature: 230°C, quadrupole temperature 150°C.

### qRT-PCR

Gene expression levels in fruit peel of different varieties were determined by quantitative real-time PCR (qRT-PCR) using SYBR Green qPCR Mix (Takara, Japan) on the CFX96 Real-Time PCR System (BIO-RAD, USA). Real-time RT-PCR analysis was carried out in a total volume of 20 μl, containing 9 μl of 2×SYBR Green qPCR Mix (Takara, Japan), 0.1 μM of each specific primer, and 100 ng of template cDNA. The reaction mixtures were heated to 95°C for 30s, followed by 39 cycles at 95°C for 10s, 59°C for 15s, and 72°C for 30s. The differences in gene expression were calculated using the 2^−ΔΔCt^ analysis method, and the transcription levels were determined by relative quantification using the Malus 18S ribosomal RNA gene as the reference gene. The primers for qRT-PCR were designed as described in S1 Table.

### Scan electric light microscopy

For light microscopy, peel tissue samples were fixed and embedded in wax as described previously. Sections were cut to a 5mm*5mm cube, and placed on glass slides.

### Y1H assays

A Y1H system was used to study the transcriptional activation by the McWRI1 transcription factor. The open reading frame of McWRI1 was cloned into the BamHI and EcoRI sites of the pGADT7 vector (Clontech), this served as an effector construct. The predicted function element ‘G-box & GCC-box’ in *McKCS* promoter sequences, ‘GCC-box & CAACA’ of *McLAC* promoter sequences and the complete promoter sequences of *McWAX* were inserted upstream of the AbAi reporter gene in a pAbAi vector. And the Y1Hgld [pMutant & GCC-box-AbAi]、Y1Hgld [pG-box & Mutant-AbAi]、Y1Hgld [pMutant & CAACA-AbAi]、Y1Hgld [pGCC-box & Mutant-AbAi] and Y1Hgld [p53-AbAi] act as the control. The effector and reporter or control constructs were transformed into competent cells of the yeast strain Y1Hgld, which resulted in the following yeast strains: Y1Hgld [pG-box & GCC-box AbAi]-pGADT7-WRI1、Y1Hgld [pMutant & GCC-box-AbAi]-pGADT7-WRI1、Y1Hgld [pG-box & Mutant-AbAi]-pGADT7-WRI1、Y1Hgld [pGCC-box & CAACA-AbAi]-pGADT7-WRI1、Y1Hgld[pMutant & CAACA-AbAi]-pGADT7-WRI1、Y1Hgld [pGCC-box & Mutant-AbAi]-pGADT7-WRI1、Y1Hgld [pLAC-AbAi]-pGADT7-WRI1、Y1Hgld[p53-AbAi]-pGAD-Rec-p53. The cells were selected on the media supplemented with leucine and Aureobasidin A (AbA) in optimal concentration (SD/-Leu/800ng AbA, SD/-Leu/1000ng AbA). The bacteria solution were spotted onto culture plates at 30°C for 3 days to confirm growth conditions.

### Statistical analysis

All data were analyzed using one-way ANOVA, followed by T test to compare differences between treatment at P<0.05 (Microsoft Excel 2010, Statistical Product and Provisional Service Solutions (SPSS v19.0) and Origin v7.05).

## Results

### Cloning of the full-length *McWRI1*cDNA and the expression differences among varieties of *Malus* plants

**Fig 1:**
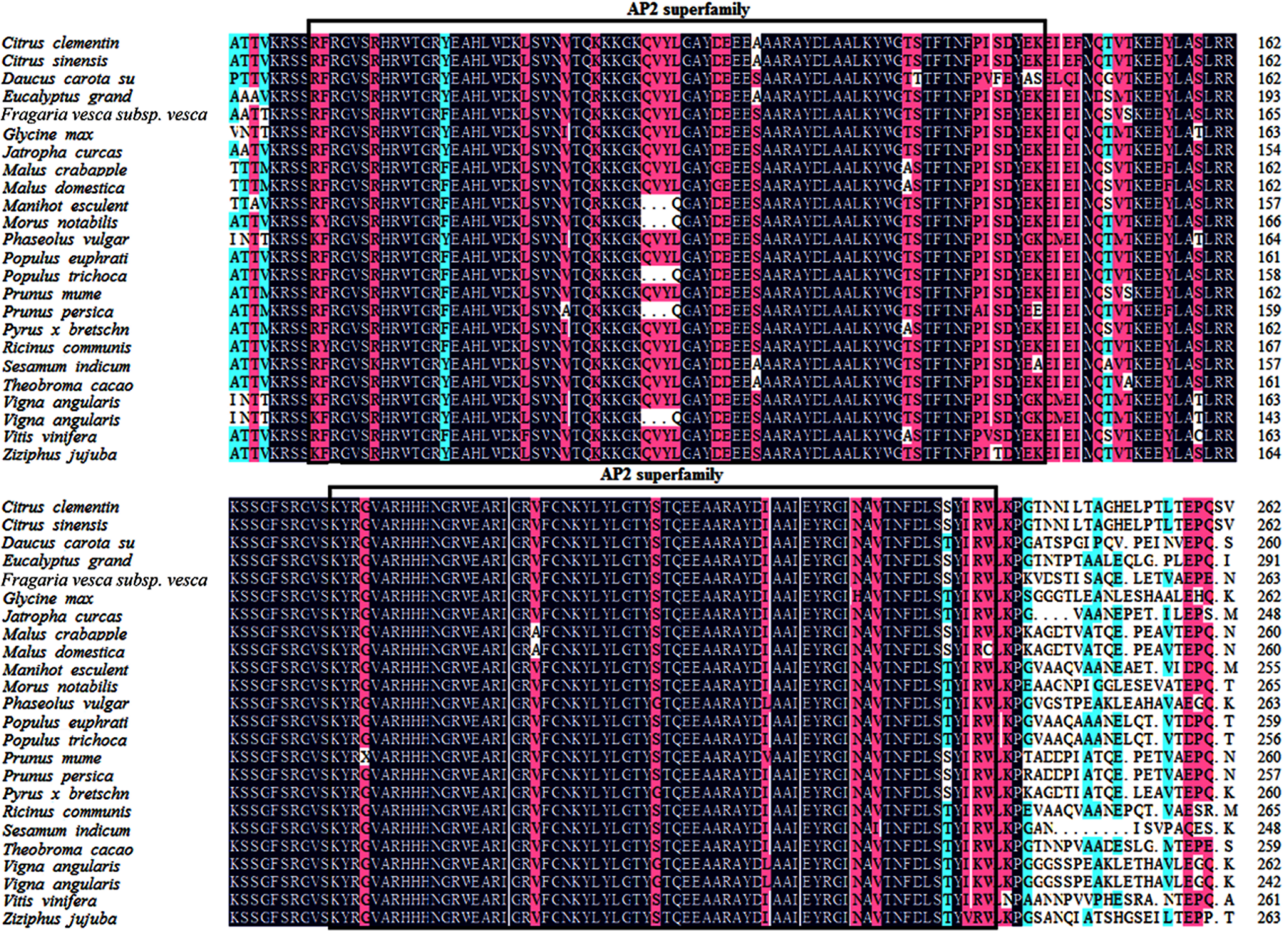
Sequence analysis of WRI1 homologues. Alignments of two domains shown to be functionally relevant to this clade are displayed, with the functionally critical valine residue indicated with a black box.

Based on homology with *Malus* domestic sequences and related sequences in NCBI, we designed a pair of oligo nucleotide primers for *McWRI1* gene cloning. Analyses of the deduced amino acid sequence revealed that *McWRI1* has 1228 bp and encodes the polypeptide which include 418 amino acids, and the polypeptide contain two AP2 conserved domain (70–118, 140–196) which consist of 48 and 58 amino acids. *McWRI1* belongs to the AP2 family of AP2/EREBP by analysis the sequence of conservation region (Fig 1).

**Fig 2:**
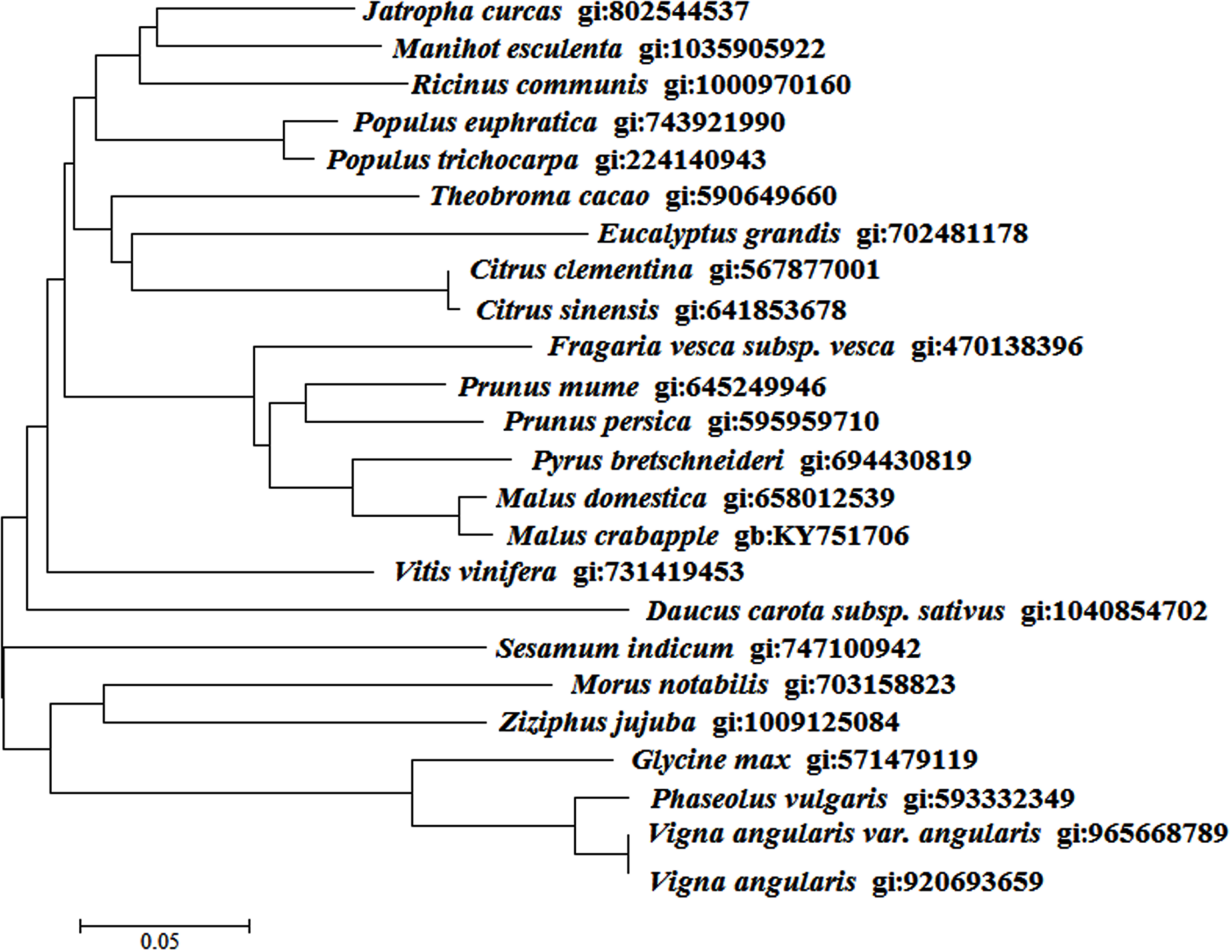
*WRI1* homologues molecular phylogeny. Molecular phylogeny of characterized members of the *McWRI1* clade. Multiple alignments of amino acid sequences were performed using DNAMAN 7. A phylogenetic tree was then constructed by the neighbor-joining method with MEGA 7.

To further explore the molecular evolutionary relationships of *McWRI1* with WRI1 protein sequences from other varieties, a phylogenetic tree was constructed using MEGA 5.10 (with the minimum evolution phylogeny test and 1,000 bootstrap replicates). We inferred that *McWRI1* may have a related function to the other of these known *WRI1* genes showed in Fig 1, and due to the high degree of similarity with *MdWRI1*, and we believe that *McWRI1* has the same function characteristic (Fig 2).

**Fig 3:**
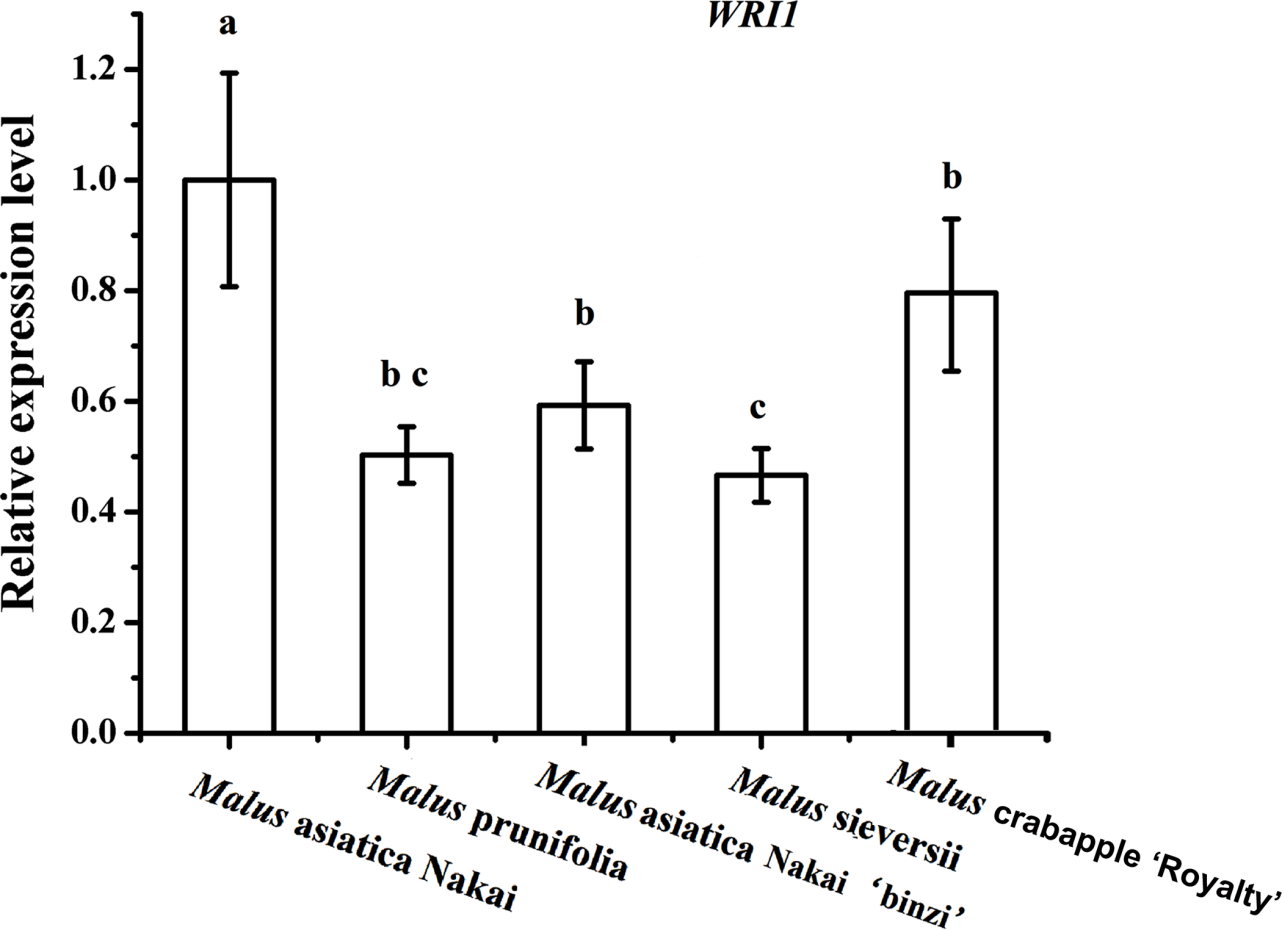
*McWRI1* expression level in the fruits of *Malus asiatica* Nakai, *Malus* prunifolia, *Malus* asiatica Nakai ‘binzi’, *Malus sieversii and Malus* crabapple ‘Royalty’. The error bars represent the mean ± SD of three biological replicates. Differences from the control samples were determined using Duncan’s test: P ≤ 0.05.

We test *WRI1* expression level among different *Malus* varieties which are *Malus asiatica* Nakai, *Malus* prunifolia, *Malus asiatica* Nakai ‘binzi’, *Malus sieversii* and *Malus* crabapple ‘Royalty’, and their growth are all under normal environmental conditions, sampling the fruit peels of these 5varieties. The conspicuous difference of *WRI1* gene expression level is between *Malus* asiatica Nakai and *Malus* sieversii, 1 and 0.48 (Fig 3). Thus, we decided to take these two types as material to continue the subsequent study.

### Low temperature stress alters characteristic of the epicuticular wax in *Malus* crabapple

**Fig 4:**
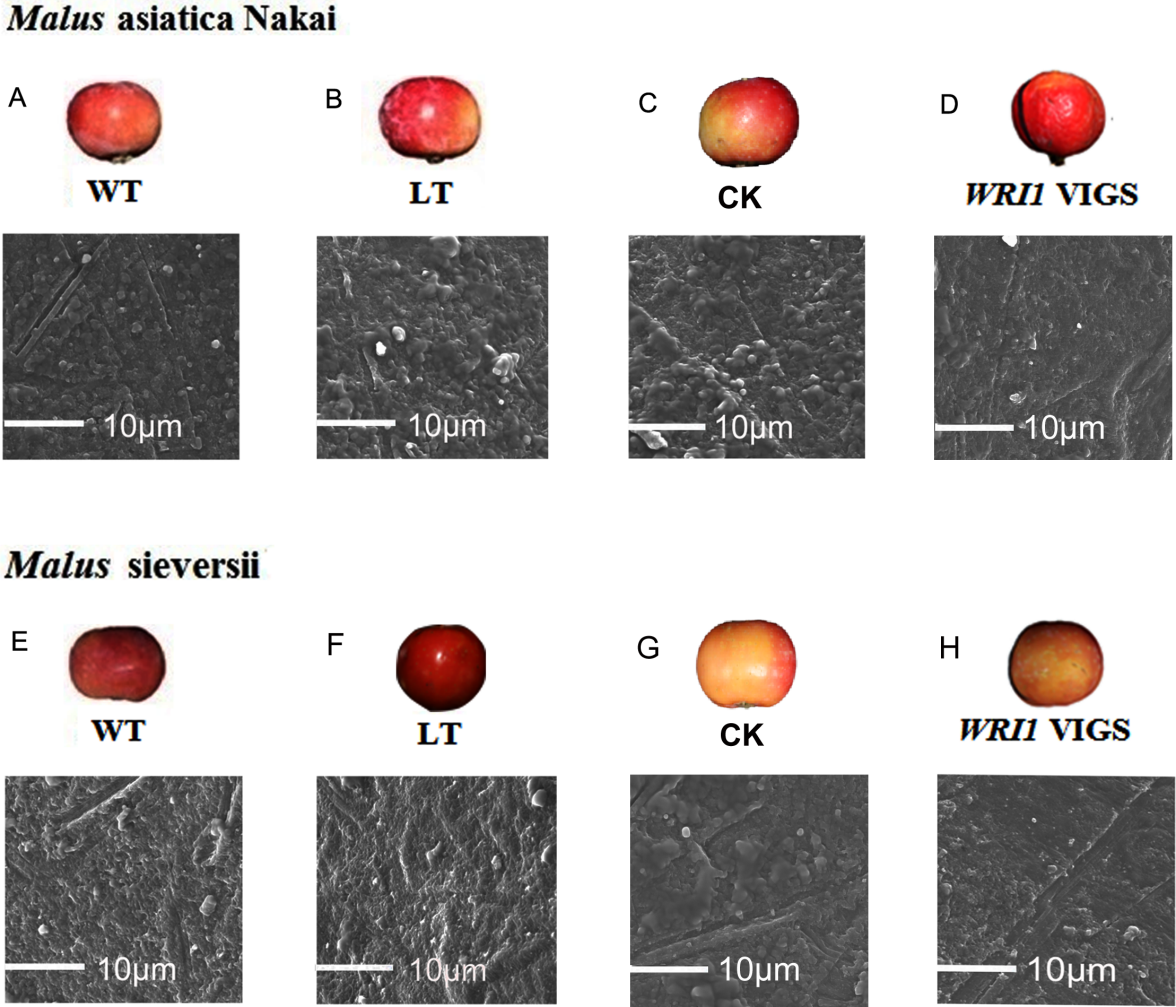
Description of fruits anatomic structures of low temperature and *WRI1* silencing group compared with wild type. A,B,C,D. Phenotypes and light microscopy image of *Malus asiatica* Nakai after low temperature and *WRI1* silencing stimulating compared with wild type and CK. E, F, G, H. Phenotypes and light microscopy image of *Malus sieversii* after low temperature and *WRI1* silencing stimulating compared with wild type and CK.

We observed epicuticular wax in the surface of two varieties fruits and found the distribution of fragments wax on the surface of *M*. asiatica Nakai fruits, and distribution of irregular mesh wax on the surface of *M*. Sieversii fruits, which showed higher thick and dense wax layer and fruit powder of the outside surface in *M*. asiatica Nakai fruits peel than those in *M*. Sieversii fruit peel, and the latter had thicker and denser wax layer in internal surface of epicuticular. Low temperature induced the increase of thickness of wax layer in two varieties fruit peel, and increased the thickness and denseness of fruit powder in the outside surface of *M*. asiatica Nakai fruits and *M*. Sieversii fruits, and it exhibited more wrinkles and irregular bulges in *M*. Sieversii fruits (Fig 4).

**Fig 5:**
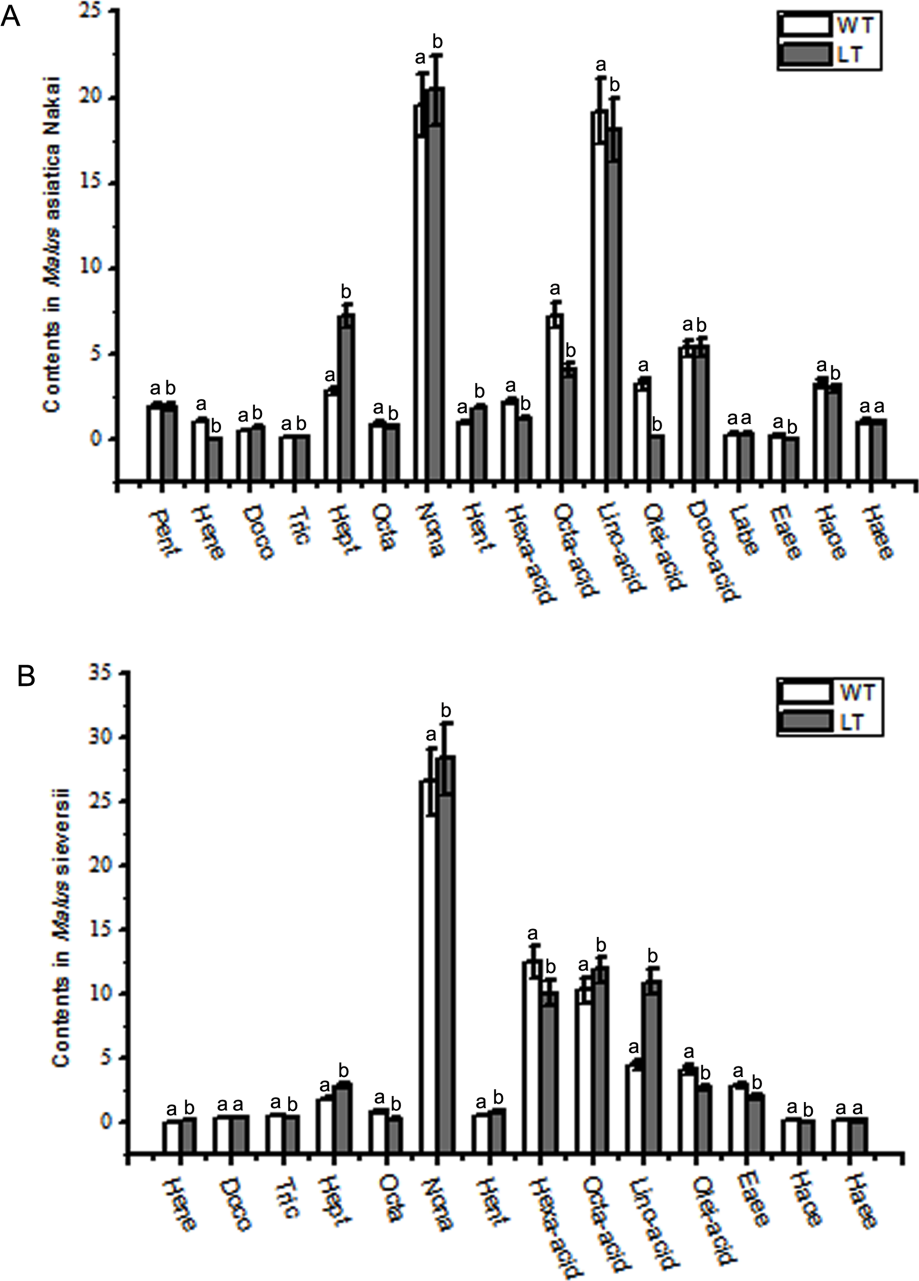
Main components of cuticular wax under low temperature stimulated condition by GC-MS. A. The contents of pentadecane (Pent), heneicosane (Hene), docosane (Doco), tricosane (Tric), heptacosane (Hept), octacosane (Octa),nonacosane (Nona), hentriacontane (Hent), hexadecanoic acid (Hexa-acid), octadecanoic acid (Octa-acid), linoleic acid (Lino-acid), oleic acid (Olei-acid), docosanoic acid (Doco-acid), linoleic acid butyl ester (Labe), erucic acid ethyl ester (Eaee), hexadecanoic acid—octadecane ester (Haoe), hexadecanoic acid—eicosane ester (Haee) in *Malus asiatica* Nakai. B. The contents of henicosane, docosane, tricosane, heptacosane, octacosane, nonacosane, hentriacontane, hexadecanoic acid, octadecanoic acid, linoleic acid, oleic acid, erucic acid ethyl ester, hexadecanoic acid—octadecane ester, hexadecanoic acid—eicosane ester in *Malus sieversii*.

The components of epicuticular wax identified by GC-MS analysis presented in Table 1. It was seen that the main compounds of epicuticular wax in surface of *Malus* crabapples fruits were included in kinds of alkanes, fatty acid and esters. In the fruits of *M*. asiatica Nakai, when compared with WT groups, the relative contents of the main components, including docosane, heptacosane, nonacosane, hentriacontane, and docosanoic acid, significant increased, while the relative contents pentadecane, heneicosane, hexadecanoic acid, octadecanoic acid, linoleic acid, oleic acid, erucic acid ethyl ester, and hexadecanoic acid—octadecane ester decreased under low temperature, with that, the relative contents of tricosane, octacosane, linoleic acid butyl ester, and hexadecanoic acid—eicosane ester appeared little differences between treatments (Fig 5A). In fruits of *M*. Sieversii under low temperature the relative contents of heptacosane, nonacosane, hentriacontane, octadecanoic acid, and linoleic acid displayed significant higher level, and the relative content of tricosane, octacosane, hexadecanoic acid, oleic acid, and erucic acid ethyl ester were significant lower than those in WT groups, while the relative contents of heneicosane, docosane, hexadecanoic acid—octadecane ester and hexadecanoic acid—eicosane ester appeared little differences with different treatment (Fig 5B). It was worthy to note that the relative content of nonacosane in fruits surface of two varieties were significant inclined under low temperature.

**Table 1:** Main components content of cuticular wax under low temperature and VIGS stimulated.

Class	Component	WT (%)	LT (%)	CK (%)	*wri1* (%)
*Malus* asiatica Nakai	Alkanes	23.54	31.05	25.57	34.58
	Organic acid	40.88	28.95	40.88	36.64
	Esters	4.68	4.33	4.68	4.43
*Malus* sieversii	Alkanes	29.87	33.08	29.87	33.29
	Organic acid	39.97	35.71	39.97	29.81
	Esters	3.12	2.23	3.12	1.82

**Fig 6:**
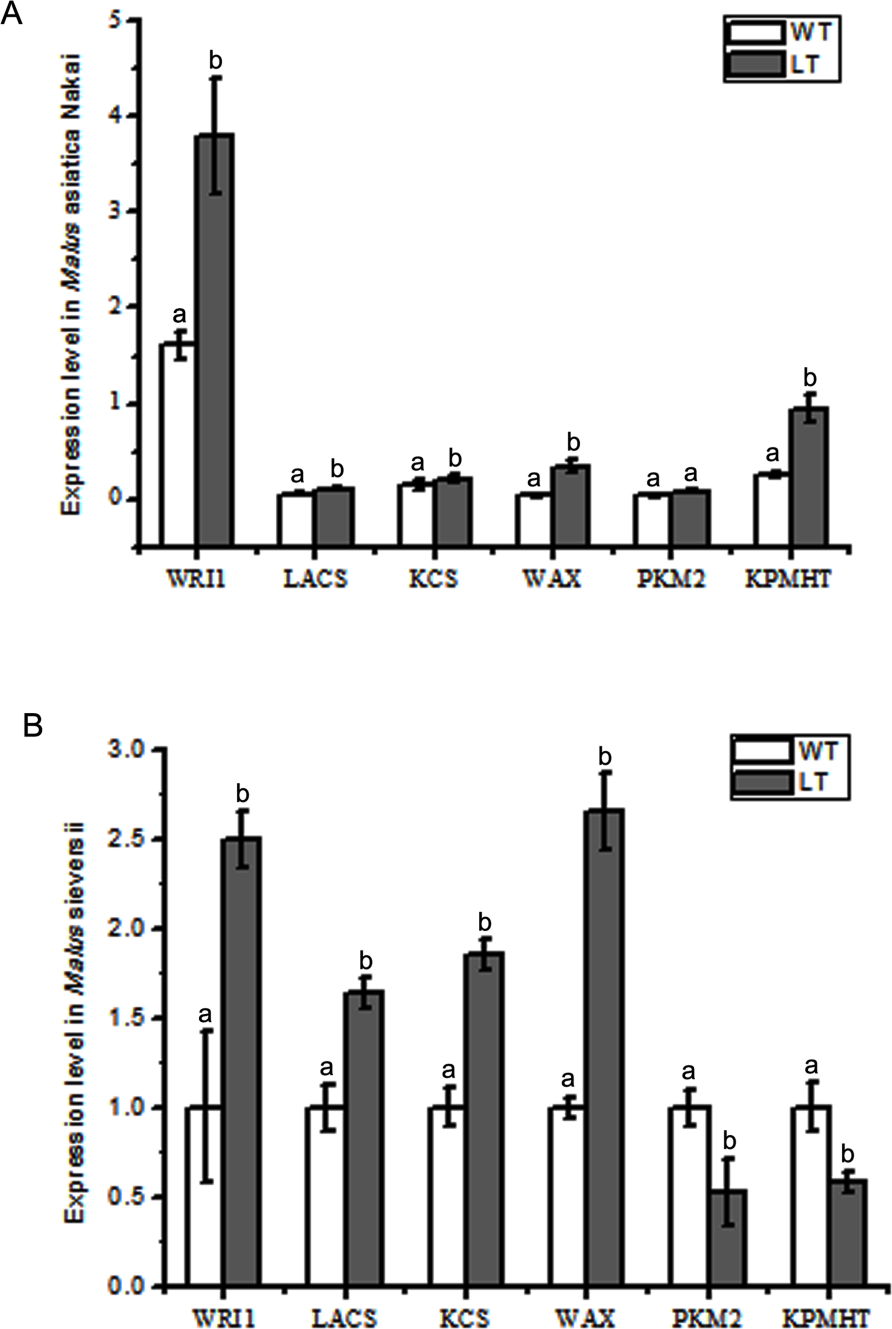
Comparison of relative gene expression level under low temperature stimulated and room temperature conditions in the fruits of two classes. A. The expression of *McWRI*, *McLACS*, *McKCS*, *McWAX*, *McPKM2* and *McKPMHT* in *Malus asiatica* Nakai. B. The expression of *McWRI*, *McLACS*, *McKCS*, *McWAX*, *McPKM2* and *McKPMHT* in *Malus sieversii*. The error bars represent the mean ± SD of three biological replicates. Differences from the control samples were determined using Duncan’s test: P ≤ 0.05.

Based on the different phenotype and main components changes in the epicuticular wax presenting between two treatments, we demonstrated that there were differences in gene expression related the epicuticular wax biosynthesis, where, 6 relevant genes were detected. It showed that low temperature up-regulated the expression *McLACS*, *McKCS*, and *McWAX* in the fruits epicuticular wax of two varieties, while the *McPKM2* and *McKPHMT* expression in the fruits epicuticular wax of *Malus* asiatica Nakai were upregulated and were down-regulated in the fruits epicuticular wax of *Malus* sieversii (Fig 6). It was important that low temperature induced significantly *McWRI1* expression level increasing, which is possible to result in the higher expression of *McLACS*, *McKCS*, and *McWAX*, related biosynthesis of epicuticular wax. However, the different relations of *McWRI1* and *McPKM2* /*McKPHMT* expression in fruits surface of two varieties indicated distinct response to low temperature stress.

### *McWRI1* Silencing alters characteristic of the epicuticular wax in *Malus* crabapple fruits

We also observed the changes of the epicuticular wax in the surface of two varieties fruits after *McWRI1*-VIGS silence. Result was found that the epicuticular wax on the surface of two fruits treated with VIGS silence appeared less than those in CK. In *M*. asiatica Nakai fruits silenced *McWRI1* by VIGS the most fragments of epicuticular wax changed into a flat circular with distribution of evacuation. In *M*. Sieversii fruits silenced *McWRI1* by VIGS the mesh wax changed into more irregular intensive with some cracks (Fig 4).

**Fig 7:**
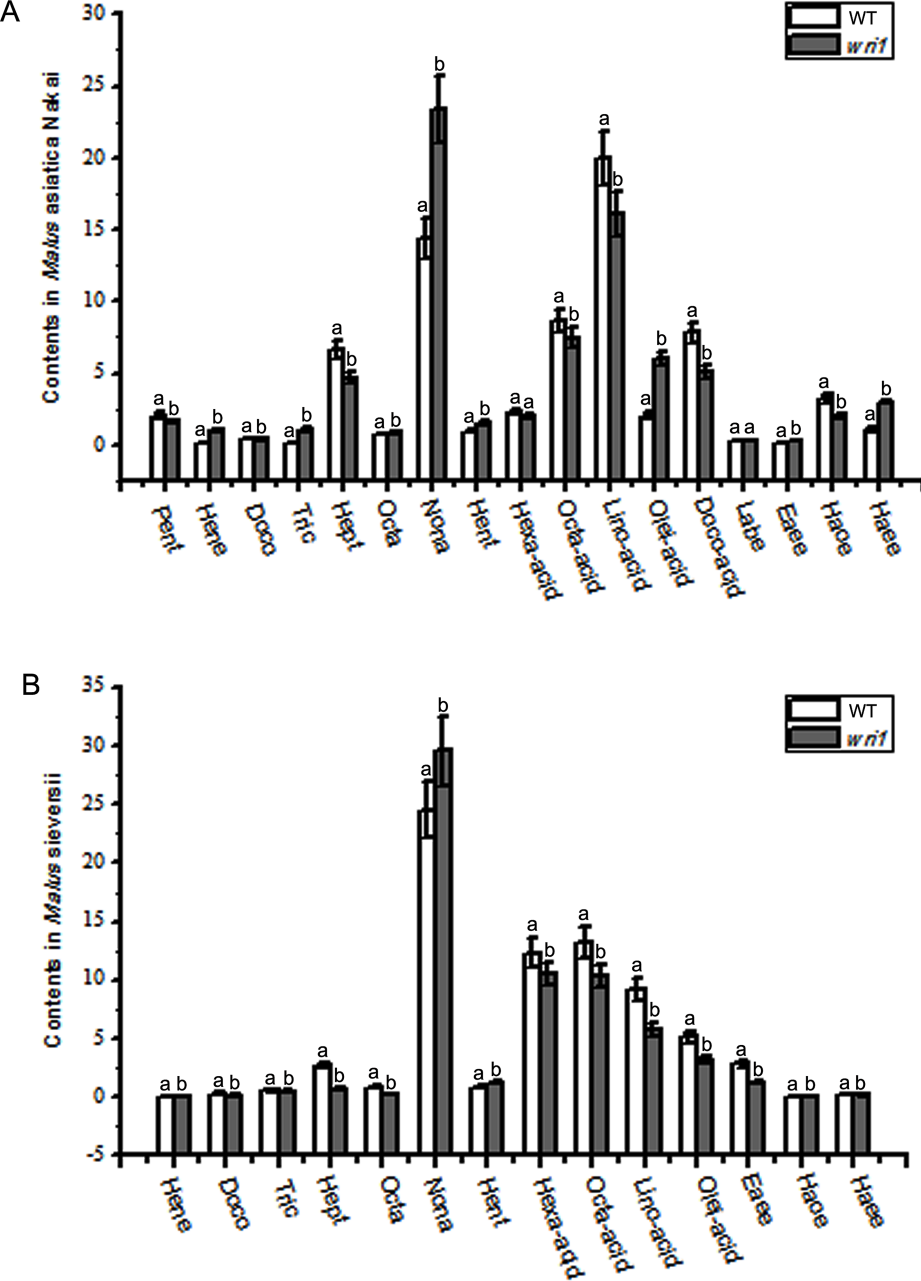
Main components analysis of cuticular wax between *WRI1* silencing group and wild type by GC-MS. A.The contents of pentadecane (Pent), heneicosane (Hene), docosane (Doco), tricosane (Tric), heptacosane (Hept), octacosane (Octa), nonacosane (Nona), hentriacontane (Hent), hexadecanoic acid (Hexa-acid), octadecanoic acid (Octa-acid), linoleic acid (Lino-acid), oleic acid (Olei-acid), docosanoic acid (Doco-acid), linoleic acid butyl ester (Labe), erucic acid ethyl ester (Eaee), hexadecanoic acid—octadecane ester (Haoe), hexadecanoic acid—eicosane ester (Haee) in *Malus asiatica* Nakai. B. The contents of henicosane, docosane, tricosane, heptacosane, octacosane, nonacosane, hentriacontane, hexadecanoic acid, octadecanoic acid, linoleic acid, oleic acid, erucic acid ethyl ester, hexadecanoic acid—octadecane ester, hexadecanoic acid—eicosane ester in *Malus sieversii*.

The components of epicuticular wax identified by GC-MS analysis presented in Table 1. It was found that the main compounds of epicuticular wax in surface of *Malus* crabapples fruits were included in three kinds of alkanes, fatty acid and esters. In the fruits of *M*. asiatica Nakai, when compared with CK groups, the relative contents of the main components, including heneicosane, tricosane, octacosane, nonacosane, hentriacontane, oleic acid, hexadecanoic acid—eicosane ester, significant increased, while the relative contents of pentadecane, heptacosane, hexadecanoic acid, octadecanoic acid, linoleic acid, docosanoic acid, and hexadecanoic acid—octadecane ester decreased after *wri1* silencing treatment, with that, the relative contents of docosane, Linoleic acid butyl ester and erucic acid ethyl ester appeared little differences between treatments (Fig 7A). In the fruits of *M*. Sieversii with *wri1* silencing, the relative contents of nonacosaneand, hentriacontane displayed significant higher level, and the relative content of docosane, tricosane, heptacosane, octacosane, hexadecanoic acid, octadecanoic acid, linoleic acid, oleic acid and erucic acid ethyl ester were significant lower than those in CK groups, while the relative contents of heneicosane, hexadecanoic acid—octadecane ester and hexadecanoic acid—eicosane ester appeared little differences with different treatments (Fig 7B). It was seen that the relative content of nonacosane in fruits surface of two varieties were significant inclined under *wri1* silencing treatment.

**Fig 8:**
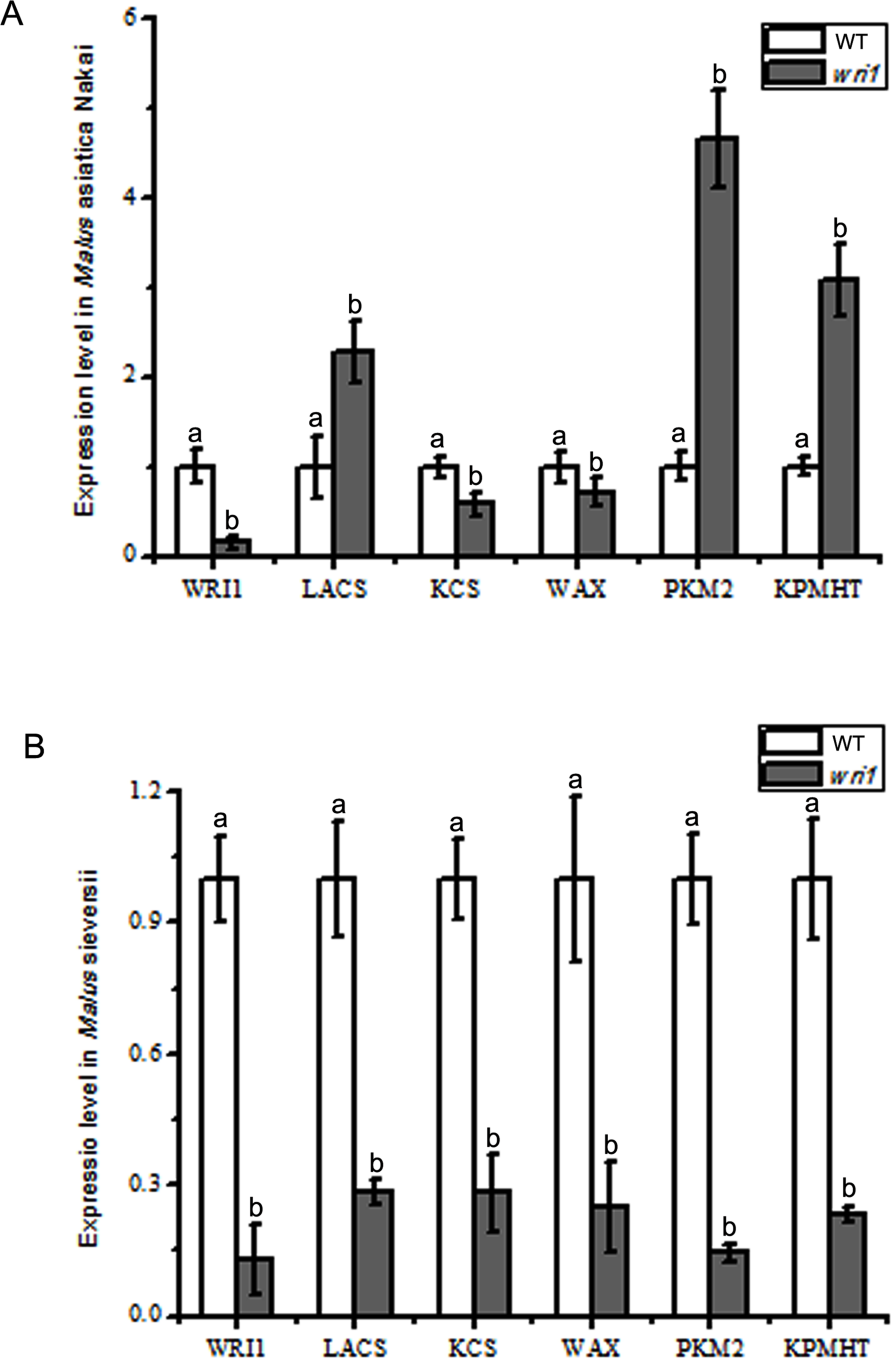
Comparison of relative gene expression level between wild type and *McWRI1* silencing in the fruits of two classes. A. The expression of *McWRI*, *McLACS*, *McKCS*, *McWAX*, *McPKM2* and *McKPMHT* in *Malus asiatica* Nakai. B. The expression of *McWRI*, *McLACS*, *McKCS*, *McWAX*, *McPKM2* and *McKPMHT* in *Malus sieversii*. The error bars represent the mean ± SD of three biological replicates. Differences from the control samples were determined using Duncan’s test: P ≤ 0.05.

Based on the different phenotype and main components changes in the epicuticular wax presenting between *WRI1*-silence lines and CK, we demonstrated that there were differences in gene expression related to the epicuticular wax biosynthesis, similarly, 6 relevant genes were detected. Where, *wri1* silencing down-regulated the expression *McLACS*, *McKCS*, and *McWAX* in the fruits epicuticular wax of two varieties, except that the expression of *McLACS* rise in fruit peel of the *Malus*asiaticaNakai. While the *McPKM2* and *McKPHMT* expression in the fruits epicuticular wax of *Malus*asiaticaNakai were upregulated and were down-regulated in the fruits epicuticular wax of *Malus*sieversii (Fig 8). It was equally important that *wri1* silencing induced significantly *McWRI1* expression level decreasing, resulting in the lower expression of *McLACS*, *McKCS*, and *McWAX*, related synthesis of epicuticular wax.

### McWRI1 binds to the promoter of *McKCS*, *McLAC* and *McWAX* and activates the expression of these genes

**Fig 9:**
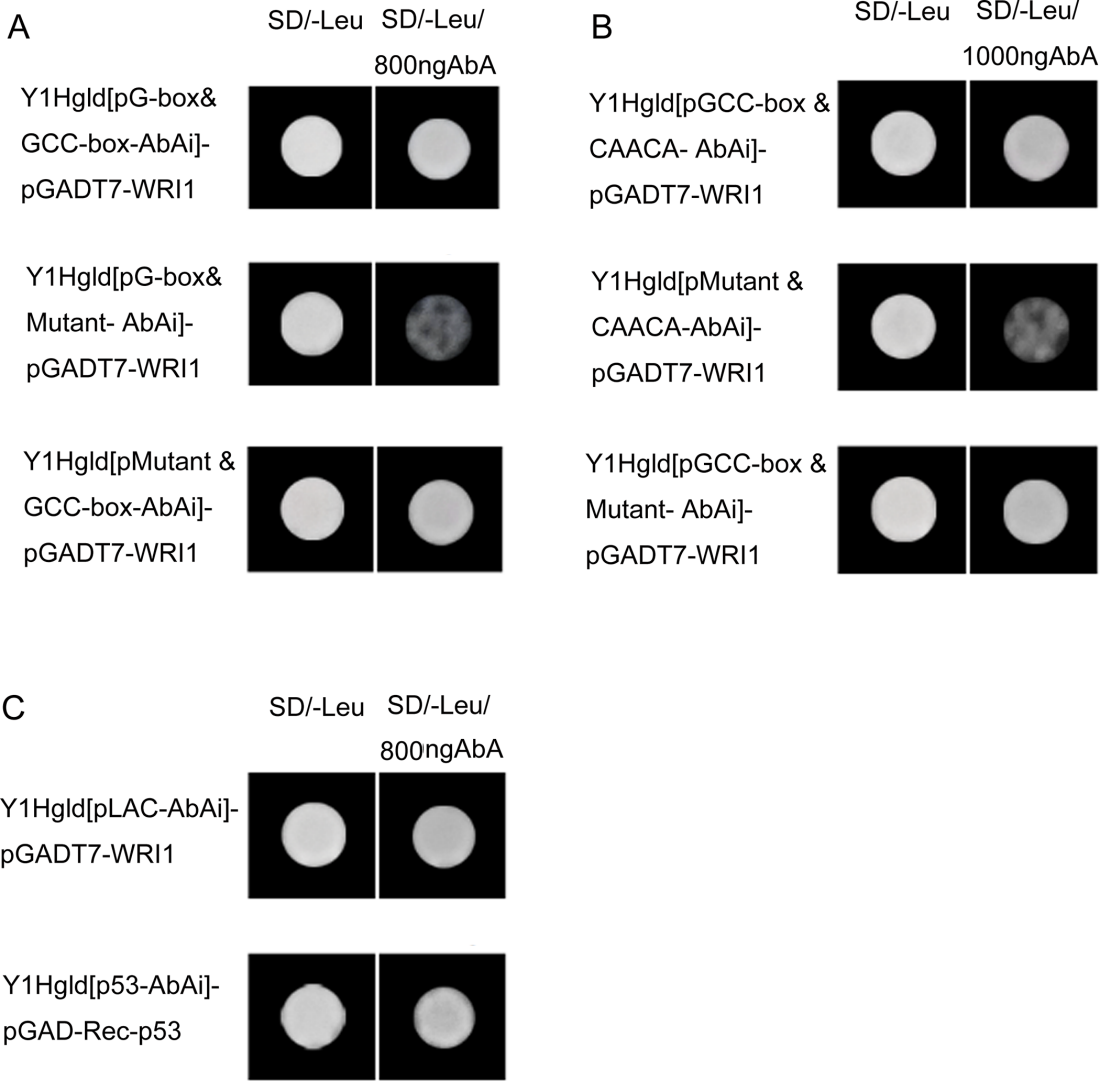
Y1H assay between McWRI1 and the promoter of *McKCS*, *McLAC* and *McWAX* genes. A.The growth of the yeast strains of *McKCS* promoter, Y1Hgld [pG-box & GCC-box AbAi]-pGADT7-WRI1,Y1Hgld [pG-box & Mutant-AbAi]-pGADT7-WRI1 and Y1Hgld [pMutant & GCC-box-AbAi]-pGADT7-WRI1on the media supplemented with leucine and Aureobasidin A (AbA) in 800ng. B. The growth of the yeast strains of the *McWAX* promoter, Y1Hgld [pGCC-box & CAACA-AbAi]-pGADT7-WRI1,Y1Hgld [pMutant & CAACA-AbAi]-pGADT7-WRI1 and Y1Hgld [pGCC-box & Mutant-AbAi]-pGADT7-WRI1 on the media supplemented with leucine and Aureobasidin A (AbA) in 1000ng. C. The growth of the yeast strains of the *McWAX* promoter, Y1Hgld [pLAC-AbAi]-pGADT7-WRI1 and Y1Hgld [p53-AbAi]-pGAD-Rec-p53on the media supplemented with leucine and Aureobasidin A (AbA) in 800ng.

To further explore the interaction between McWRI1 transcription factor and the structural genes in the biosynthesis pathway of long chain fatty acid on surface of apple fruits, we performed yeast one-hybrid (Y1H) assays. The open reading frame of McWRI1 was cloned into the pGADT7 vector, this served as an effector construct. The predicted function element were inserted upstream of the AbAi reporter gene in a p AbAi vector. And the Y1Hgld [pMutant & GCC-box-AbAi]、Y1Hgld [pG-box & Mutant-AbAi]、Y1Hgld [pMutant & CAACA-AbAi]、Y1Hgld [pGCC-box & Mutant-AbAi] and Y1Hgld[p53-AbAi] act as the control. For the KCS promoter, the result showed thatthe yeast strainsY1Hgld [pG-box & GCC-box AbAi]-pGADT7-WRI1 and Y1Hgld [pMutant & GCC-box-AbAi]-pGADT7-WRI1could grow normally, but the growth of yeast strainY1Hgld [pG-box & Mutant-AbAi]-pGADT7-WRI1 was inhibited by Aureobasidin A (Fig 9A). For the WAX promoter, the yeast strainsY1Hgld [pGCC-box & CAACA-AbAi]-pGADT7-WRI1 and Y1Hgld [pGCC-box & Mutant-AbAi]-pGADT7-WRI1 grew normally, but the yeast strains Y1Hgld[pMutant & CAACA-AbAi]-pGADT7-WRI1was inhibited to grow (Fig 9B). At the same time, the yeast strainsY1Hgld [pLAC-AbAi]-pGADT7-WRI1 grew as well as the yeast strains Y1Hgld [p53-AbAi]-pGAD-Rec-p53 (Fig 9C). These data further demonstrated that McWRI1 can bind to the promoter element ‘GCC-box’of the structural genes *McKCS* and *McWAX* to regulate their expression and affect the biosynthesis of long chain fatty acid. Moreover, McWRI1 interacted with the complete promoter sequence of *McLAC*, and it may activate some element in the *McLAC* promoter sequence and regulate gene expression. The special element of *McLAC* promoter will be analyzed further.

## Discussion

Cuticular wax plays a key role in plant as its multiple functions to guarantee the plant quality by limiting non-stomatal water loss [35], prevening UV radiation [36], repelling bacterial [37]. These always benefit to the preservation of the fruit storage and prolong shelf life. Previous studies suggested that cuticular wax accumulation increased after drought stress in plant including wheat, rice, alfalfa and tobacco [38–44]. In wheat the cuticular wax layer of flag leaf is obviously much denser under drought stress compared with normal condition ones [45]. Gauvrit et al revealed that there is 29% decreased of wax under low temperature in maize. Our results indicated that both low temperature and *McWRI1* silence by VIGS changed the irregular accumulation of cuticular wax and thickness and denseness distribution on fruit peel, though these were different structures and change ranges between two varieties fruits, revealing the protection of the cuticular wax to water loss in post-harvest fruits.

The structure and configuration of cuticular wax are associated closely with its chemical components on the surface of the fruits. Under environment stress, the whole cuticular wax content in *Arabidopsis* shows to go up trend, especially the main component alkane which account for majority percentage of total cuticular wax about was 70% to 80% [46]. The cuticular wax and long chain alkane are inclined increase in the leaves of Rosa rugosa under drought stress [47]. The cuticular wax component and structure of Brassica oleracea leaves were higher under low moisture than under high moisture environment [48]. The density of wax crystalloid went down and its single crystalloid shrank, and the components are also changed, primary alcohole present incline trend, while aldehydes and ketone content show decline trend under drought stress in *Arabidopsis*. Nonacosane and nonacosanol are the main component of cuticular wax in *Malus* and different varieties has different content [49]. Mazliak reported that the surface wax content on ‘Calville Blanc’ apple fruits was on a rise in the first 3 months of storage at 4°C, then a downward trend in the process of storage. Mazliak [50] also found that the surface wax components on ‘Cox Orange’ apple peel increased in first a month stored at 4°C and decreased in the process of the subsequent storage. In our study, the total contents of alkane in two cultivars are 26.71% and 28.4% on average (Table 1). Low temperature induced the obvious accumulation of most alkane components including C15, C22, C27, C29, C31 alkane (C23, C28 were induced decrease), and reduced the content of the detected lipid acids and esters in cuticular wax composition on two kind of fruit peel (except C18 alkanoic acid and linoleate in *Malus*sieversii fruits and C22 alkanoic acid in *Malus*asiaticaNakai fruits) (Fig 5). These results could indicate that low temperature stimulate the alkane-forming pathway resulting in the formation of very long chain alkanes and their derivatives on the surface of plants and the fruit peel.

The biosynthesis of the cuticular wax on plants surface include forming of C16 / C18 saturated fatty acid, synthesis of very long chain fatty acids (VLCFAs) synthesis of the wax components from VLCFAs, These steps has been known to be regulated partly by several key genes, such as *KCS*, *LACS*, *WAX*, *PKM2*, *KPHMT*, in their branches of biosynthesis, resulting in changes of structure, configuration and components of cuticular wax on the surface of plants and fruits. When plant is confronted with environment stress, these genes undertake the function of regulation to biosynthesis of cuticular wax. Ni Yu et al reported low temperature induced plant can up-regulate the expression of *CER1* to promote the alkane synthesis and up-regulated expression of *CER4* promoted the synthesis of primary alcohol under cold stress, while the down-regulated expression of *KCS1*, *CER3* and *WIN1* under cold stress indicated that the increase of total wax was due to the promotion of the alkane-synthesizing branch in *A*. thaliana plants. Jetter and Kunst reported the alkane-forming pathway resulted in the formation of very long chain alkanes and their derivatives and further promoted cuticular wax in Arabidopsis [51]. In this study, we found that low temperature induced LACs, KCS expression rise resulting in alkanes component such as nonacosane and hentriacontane significant incline, which provides the preparation in the cuticular wax for subsequent synthesis (Figs 5 and 6). WAX2 was found and identified in Arabidopsis that acted to composition and structure of cuticular wax, and associated with the non-stomatal evaporation on plant surface, study has demonstrated that WAX2 express specifically in the meristem of the stem epidermis, the young leaves and the fruit cell during early development, which was positive response to the environment signals of light, humidity and temperature. Our results indicated low temperature promoted the higher expression of *McWAX* in the fruit peel, resulting in the alkanes components increased, and the unsaturated fatty acid and ester components decreased. *McPKM2* and *McKPHMT* are associated with CoA synthesis, which play the part of the precursor of CoA in the cuticular wax on plant surface. Low temperature up-regulated their expression in fruit peel of *M*. asiatica Nakai, down-regulated their expression in the fruit peel of *M*. sieversii, which were speculated that the different varieties exhibited diversity responses to low temperature stress with the reference the comparative differences in components and content of the cuticular wax between two varieties in the present study (Fig 5).

*WRI1* is an APETALA2/ethylene-responsive element binding (AP2/EREBP) proteins, one of the largest transcription factor families [52, 53]. This family is best characterized by a common AP2 domain of about 60 amino acids that is important for DNA binding [54–57]. While the AP2/EREBP transcription factor family is unique to plants, proteins containing homologues of the AP2 domain have been identified in cyanobacteria, ciliates and viruses, where they are predicted to function as mobile endonucleases [58, 59]. It has been already proved that *WRI1* up-regulates the genes associated with fatty acid synthesis in vegetative tissues [60, 61], such as *CER1*, *KCS1* and *CER2*, these genes are regulated by WRI1 and enhance some genes which take part in wax biosynthesis. These are benefit for *Petunia*, *Arabidopsis*, tomato to resistant the salt, water, low temperature and oxygen-deficit stress. Our study verified that *McWRI1* regulated the transcription level of *McKCS*, *McLACS* and *McWAX*, causing the accumulation of alkanes components in the cuticular wax, and further promoting abundance of cuticular wax on the Malus fruits peel.

In order to identify the mechanism that *McWRI1* regulated expression of genes related the synthesis of cuticular wax on the fruit surface, we silenced the *McWRI1* by VIGS method. The results indicated that the expression of *McLACs*, *McKCS* and *McWAX* were down-regulated in the fruit peel silenced *McWRI1* of *Malus* sieversii, and the expression of *McLACS*, *McKCS* and *McWAX* down-regulated while that of *McLACS* were up-regulated in the fruit peel silenced *McWRI1* of *M*. asiatica Nakai (Fig 7), With that, content of the alkanes except C29 and C31, the unsaturated fatty acid and ester components were decreased in the fruit peel silenced *McWRI1* of *Malus sieversii*, and the most of wax components content displayed similar decrease, but tricosane, nonacosane, oleic acid and hexadecanoic acid—eicosane estercontent increased in the fruit peel silenced *McWRI1* of *M*. asiatica Nakai (Fig 8). In order to further analyze the interaction between McWRI1 and the genes *McKCS*, *McLAC* and *McWAX*, we carried out the Y1H experiment, and these results illustrated that the McWRI1 could regulate the expression of wax synthesis genes, such as *McLACs*, *McKCS* and *McWAX* by activating their promoter element, affecting the change of the composition and content in the cuticular wax on the fruit surface, and also explained the differences in the cuticular wax synthesis between two varieties fruits. There were some researches about the *WRI1* transgenic in different plants, including *Arabidopsis*, maize, *Lepidium* campestre and *Nicotiana* benthamiana in which the overexpression of *WRI1* yielded an incline of seed/leaf oil content [62–64]. These results revealed that the overexpression of *WRI1* (*WIN1/SHN1*) could give rise to incline of expression of the genes related the cuticular wax synthesis, including *McLACS*, *McKCS* and *McWAX*, resulting in the accumulation level of alkanes components in the cuticular wax on the surface of *Malus* fruits.

In *Arabidopsi*s, when *WRI1* (*Wrinkled1*) caused the decrease of seed oil content, the activities of some key enzymes related the glycolysis process were significant down-regulated in wri1 lines during the seed development, for example hexokinase and pyruvate kinase (*PK*, *PKMs*). The ectopic expression of *WRI1* also caused the of sugar accumulation, which induced seed oil accumulation especially accumulation of triacylglycerol in transgenic plants. Comparison analysis to *wri1-1* and WT by seeds microarray indicated that the transcription level of enzymes encoded glycol metabolism were significant down-regulated in wri1 seed. The further results revealed that *WRI1* regulated the transcription of the enzymes encoded glycol metabolism through *WRI1* could stimulate higher transcription level of the sugar induced promoter. Christoph Benning et al also found that seedlings of wri1-1 mutants had significant sensitive response to ABA and sugars. Our results indicated that low temperature and *WRI1* VIGS silence caused the transcription level of *McPKM2* and *McKPTM* significant down-regulation, and up-regulation in fruit peel of *Malus* sieversii and *Malus* asiatica Nakai, respectively. We speculated they were not only associated with the biosynthesis of the cuticular wax on the malus fruit peel, but also relative to other synthesis pathway at the same time in different varieties under different conditions.

However, the accumulation of fruit cuticular wax and its gene network is complex, which appeared diversity among cultivars/varieties under the different environment stress. For example, some studies revealed that under normal environment *WRI1* is an up-regulator in the biosynthesis of wax in *Arabidopsis*, *Lepidium* campestre, *N*. benthamiana and tobacco plants [65–67], while WRI1 is a down-regulator under low temperature in Arabidopsis, the low temperature stimulated *WRI1* decreased but *WAX* increased. *M*. asiatica Nakai and sieversi are important varieties and cross parents of the apple breeding, which composition, structure and abundance in the cuticular wax on the fruit peel displayed some differences, resulting from the mechanism that the transcription level of genes related the cuticular wax synthesis regulated by transcript factor *McWRI1* under different environment conditions, Of these interactions and regulating mechanism still needs further research.

## Supporting information

S1 Table The primer list used in experiment. (DOCX)
