# Supplementary material for: McWRI1, a transcription factor of the AP2/SHEN family, regulates the biosynthesis of the cuticular waxes on the apple fruit surface under low temperature
Source: PLoS One. 2017 Oct 26;12(10):e0186996. doi: 10.1371/journal.pone.0186996 (PMC5658121; doi:10.1371/journal.pone.0186996)
Supplement: S1 Table — (DOCX) [file pone.0186996.s001.docx]

**Supplementary table1.**

| Primer name | Primer sequence |
| --- | --- |
| 18SR-F | 5’- GTCACTACCTCCCCGTGTCA -3’ |
| 18SR-R | 5’- GAGCCTGAGAAACGGCTACC -3’ |
| McWAX-F | 5’- AAACCTCCATTCCTTCCACCT -3’ |
| McWAX-R | 5’- GCTTGATTTTGATTGGGAGGATAG -3’ |
| McKCS-F | 5’- CCAGCCACAGCCATCAAGT -3’ |
| McKCS-R | 5’- GCATATAATTGCGTATTCCAAAAG -3’ |
| McLACS-F | 5’- TACAAGTTGGCGAATCTGGAA -3’ |
| McLACS-R | 5’- GCACCTGACAACAATATACGAACT -3’ |
| McPKM2-F | 5’- ACGAGGTGATCTTGGAATGG -3’ |
| McPKM2-R | 5’- AGTGCCATCTAGAACGGCAT -3’ |
| McKPHMT-F | 5’- ATGGGGCATGTGGGACTTAC -3’ |
| McKPHMT-R | 5’- AGATGTTGCAGCAGCAGCGA-3’ |
| McWRI1-F | 5’-  AGGAGTAGCAAGGCACCATCATA -3’ |
| McWRI1-R | 5’- GGGTACTGTAAGTGCCAAGGTAGAG -3’ |
